# Systemic Lupus Erythematosus (SLE) and Thrombotic Thrombocytopenic Purpura (TTP) Presenting as Recurrent Strokes: A Case Report

**DOI:** 10.7759/cureus.60238

**Published:** 2024-05-13

**Authors:** Abhinav Kadam, Saket Toshniwal, Sourya Acharya, Samarth Shukla

**Affiliations:** 1 Department of Medicine, Jawaharlal Nehru Medical College, Datta Meghe Institute of Higher Education and Research, Wardha, IND; 2 Department of Pathology, Jawaharlal Nehru Medical College, Datta Meghe Institute of Higher Education and Research, Wardha, IND

**Keywords:** nephrotic, anemia, thrombocytopenia, proteinuria, plasmapheresis

## Abstract

Thrombotic thrombocytopenic purpura (TTP) is a rare but potentially life-threatening condition characterized by fever, acute hemolysis, thrombocytopenia, renal dysfunction, and CNS dysfunction. A peripheral smear shows schistocytes because of microangiopathy. It is extremely rare for TTP and systemic lupus erythematosus (SLE) to coexist. This report details an Indian female patient's uncommon clinical presentation of TTP linked to SLE. A 52-year-old woman presented to the emergency department with complaints of altered sensorium and weakness of the right side of the body. She was initially evaluated as a case of a cerebrovascular accident. CT brain imaging revealed multiple ischemic infarcts involving both cerebral hemispheres. MRI brain confirmed the same. During further evaluation, she was found to have hemolytic anemia, thrombocytopenia, and nephrotic range proteinuria. Immunological investigations confirmed SLE. A peripheral smear revealed schistocytes, and the PLASMIC score confirmed a high risk of TTP. The patient was treated with immunosuppressants, plasma exchange, and hemodialysis, along with other supportive measures. The patient showed a positive response to the therapy mentioned, with improved power and renal function. The patient denied a renal biopsy and was discharged after two weeks. This case report emphasizes the importance of the association between TTP and SLE.

## Introduction

Systemic lupus erythematosus (SLE) is characterized by autoantibodies that target nuclear antigens [1]. The condition may appear as a fever, skin changes, or hair loss with multisystem involvement, including the central nervous system (CNS), renal system, or hematological system [2].

Thrombotic thrombocytopenic purpura (TTP) is caused by a deficit in ADAMTS13, commonly referred to as von Willebrand factor-cleaving protease (VWFCP), which affects the capillaries and arterioles of several organs [3]. One important pathologic finding in TTP is the inhibition of spontaneous microvascular platelet clumping by VWFCP, the primary regulator of VWF size, ADAMTS13, which significantly impaired most TTP patients [4].

TTP can be inherited by the *ADAMTS13* gene mutation in the plasma or can be caused by autoimmune inhibitors in acquired situations such as infections, SLE, and neoplasms. TTP is exceptionally rare in SLE patients (<0.5%) [5]. Remarkably, decreased levels of ADAMTS13 have been observed in connective tissue illnesses, including SLE, indicating a likely etiology for the coexistence of TTP and SLE [6]. In this case report, we have highlighted a patient with SLE who presented with multiple strokes, which were complicated by TTP. Currently, no such association has been documented in the literature.

## Case presentation

A 52-year-old female presented to the emergency department of this hospital in a state of altered sensorium. The informant gave a history of clumsiness in the right arm, such that she could not hold a glass. She also had a slight deviation of the angle of her face towards the left, which was recovered within 24 hours of the onset of symptoms; the patient had not visited the hospital for those complaints and used home massage for relief. This episode was three days ago. She again developed blurring of vision in both eyes and a headache, for which the patient took a general practitioner's advice and was referred to this tertiary care hospital. There was no history of seizures, cough, abdominal pain, or diarrhea. She denied any history of systemic hypertension, diabetes mellitus, or autoimmune disease. Relatives also stated no history of any family member having any autoimmune disorder.

**Figure 1:**
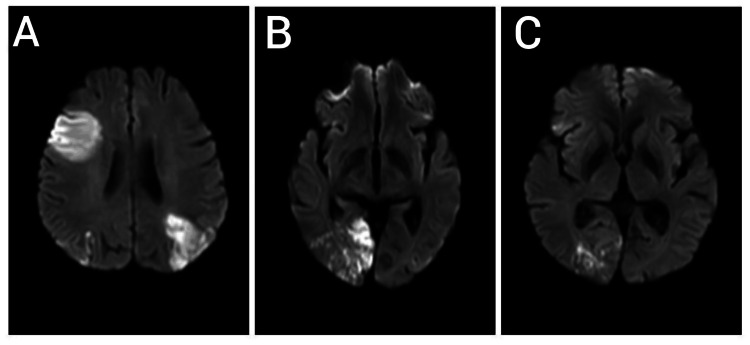
A, B, and C showing MRI brain suggestive of acute infarcts in the right frontal lobe, right insular cortex, bilateral parietal lobes, right posterior temporal lobes, and bilateral occipital lobes. MRI: magnetic resonance imaging

On examination, she was febrile, with a Glasgow Coma Scale (GCS) score of 12/15 (E4V3M5), her pulse rate was 86 beats per minute, and her blood pressure was found to be 120/70 mmHg. Oxygen saturation in room air was found to be 95%. Cardiovascular and respiratory system examinations did not yield any significant findings. There were no evident dermatological lesions on examination. Neurological examination revealed mildly altered sensorium; the patient was not moving her right upper and lower limb on painful stimulus, and an angle of mouth deviation was observed on the left side, suggesting right-sided facial weakness. Deep tendon reflexes on the right upper and lower limb were exaggerated, and the right plantar was extensor. She was immediately admitted to a medical intensive care unit with a diagnosis of a cerebrovascular episode with left hemiparesis. An urgent CT scan of the brain ruled out hemorrhage and intracerebral bleeding, and a screening MRI revealed multiple ischemic infarcts in bilateral cerebral hemispheres (Figure 1).

She was started on dual antiplatelet therapy and other supportive management. Investigations are shown in Table 1.

**Table 1:** Initial laboratory findings of the patient with an autoimmune panel with their normal reference ranges. MCHC: mean corpuscular hemoglobin concentration; MCV: mean corpuscular volume; MCH: mean corpuscular hemoglobin; RBC: red blood cell count; WBC: white blood cells; RDW: red cell distribution width; APTT: activated partial thromboplastin time; INR: international normalized ratio; SGOT: serum glutamic-oxaloacetic transaminase; SGPT: serum glutamic pyruvic transaminase; LDH: lactate dehydrogenase; ANA: antinuclear antibodies; Ds-DNA: double-stranded DNA

Laboratory Parameter	Results	Normal Values
Hemoglobin	5.2 g/dL	11-14 g/dL
MCHC	31.3 g/dL	32-36 g/dL
MCV	73.5 micron	79-92 micron
MCH	22.9 pg	27-31 pg
Total RBC count	2.27 x 10^6 ^cells/cumm	2.50-5.50 x 10^6 ^cells/cumm
Total WBC count	12000 cells/cumm	4000-11000 cells/cumm
Total platelet count	0.26 x 10^6 ^cells/cumm	1.50-4.50 x 10^6 ^cells/cumm
Hematocrit	16.7%	40%-54%
Monocyte	4%	2%-8%
Granulocyte	75%	40%-60%
RDW	16.4 fL	12.2-16.1 fL
Eosinophils	1%	1-4%
Basophil	0%	<1%
Reticulocyte count	2.8%	0.5%-2.5%
APTT	30.5 seconds	29.5 seconds
Prothrombin time	12.3 seconds	11.3 seconds
INR	1.03	1.00
Urea	81 mg/dL	6.24 mg/dL
Creatinine	7.1 mg/dL	0.59-1.04 mg/dL
Sodium	137 mEq/L	135-145 mEq/L
Potassium	6.1 mEq/L	3.5-5.1 mEq/L
Calcium	7.6 mEq/L	8.4-9.8 mEq/L
Alkaline phosphate	124 IU/L	75-124 IU/L
SGOT	23 IU/L	8-45 IU/L
SGPT	12 IU/L	7-56 IU/L
Total protein	6.8 g/dL	6.0-8.3 g/dL
Albumin	2.6 g/dL	3.4-5.4 g/dL
Total bilirubin	0.9 mg/dL	0.1-1.0 mg/dL
Conjugated bilirubin	0.2 mg/dL	0.1-0.4 mg/dL
Unconjugated bilirubin	0.7 mg/dL	0.2-0.6 mg/dL
Magnesium	2.0 IU/L	1.6-2.1 IU/L
Phosphorus	6.7 mg/dL	3-4.5 mg/dL
Serum LDH	995 U/L	140-280 U/L
Creatinine–urine	47.5 mg/dL	20-320 mg/dL
Protein–urine	293 mg/dL	0-14 mg/dL
Urine protein/creatinine ratio	6.16 mg/mg	0.15-0.50 mg/mg
24-hour urine protein	8490 mg/day	<150 mg/day
HIV card test	Negative	-
ANA by immunofluorescence	+++ speckled pattern with estimated titer 1:1000	-
Complement 3	150 mg/dL	90-180 mg/dL
Complement 4	6.3 mg/dL	10-40 mg/dL
ADAMTS13	42%	50%-160%
Ds-DNA	88 IU/mL	>75 IU/mL–Positive

**Figure 2:**
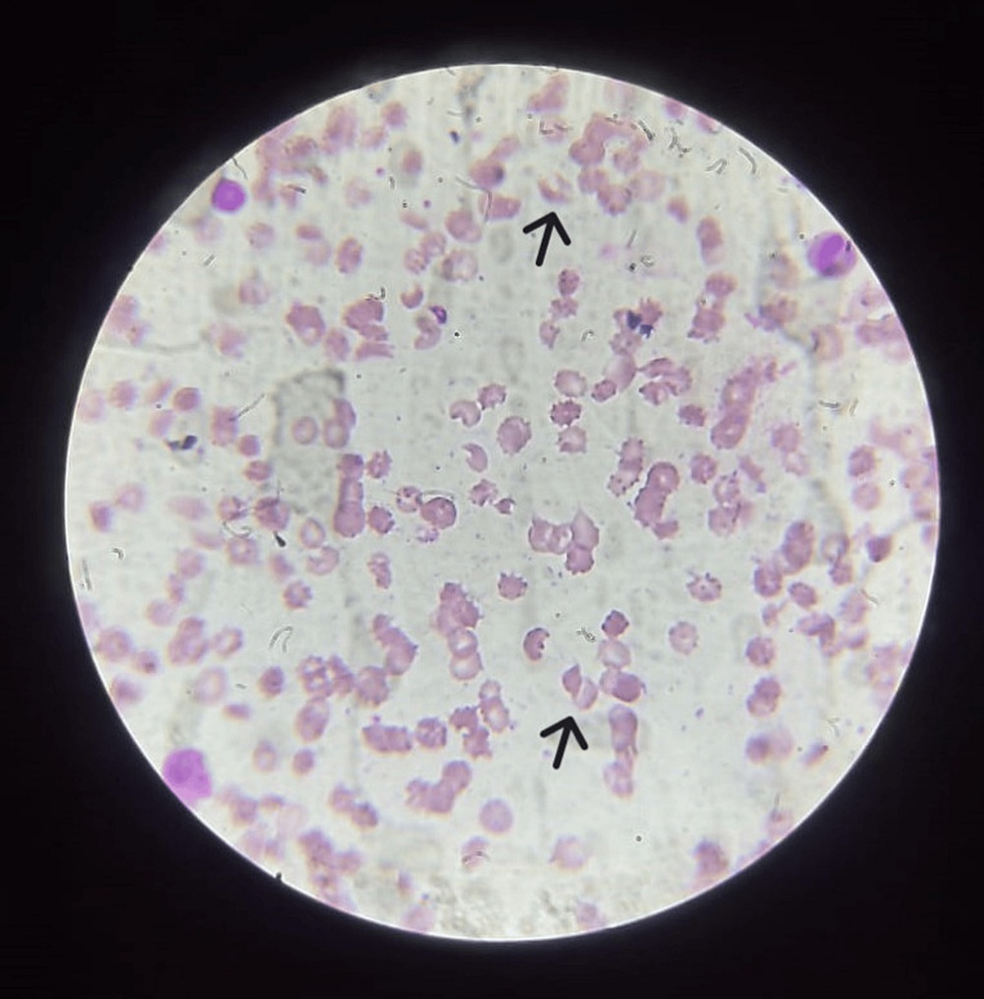
Peripheral smear with Giemsa stain (100× view) showing schistocytes (arrows).

Upon peripheral smear examination, schistocytes were found in significant amounts, leading to suspicion of a microangiopathic hemolytic state (Figure 2).

Coombs test was done and found to be negative. The autoimmune panel (Table 1) revealed positive antinuclear antibody and anti-ds-DNA status. SLE was confirmed according to the European League Against Rheumatism/American College of Rheumatology (EULAR/ACR) criteria with features such as fever, thrombocytopenia, autoimmune hemolysis, and the presence of anti-ds-DNA (score 16). The PLASMIC score was calculated to be 6 points, suggesting a high risk for TTP. Given thrombotic microangiopathy with a negative Coombs test, the ADAMTS13 test was ordered and was found to be 42%.

The blood, stool, urine, and sputum cultures were negative. The chest radiograph revealed bilateral pulmonary edema. An ultrasound of the abdomen showed mildly raised echotexture of bilateral kidneys with maintained corticomedullary differentiation in normal-sized kidneys and otherwise normal ultrasound findings. The 2D echocardiography was normal. A renal biopsy was also scheduled in light of the urine analysis findings to rule out lupus nephritis. However, it was not carried out since her relatives did not consent.

The patient was treated with intravenous methylprednisolone 1 g for three days and then switched on to oral prednisolone 60 mg through Ryle’s tube. Three units of packed red blood cells were transfused. Five hemodialysis sessions were initiated, and five sessions of plasma exchange were carried out along with higher antibiotics and supportive measures.

The patient’s consciousness improved by day six, and she started recovering. Hematological and renal profile improved. The patient was discharged after 15 days with mild residual hemiparesis with a tapering dose of corticosteroids. Follow-up after 15 days is awaited.

## Discussion

TTP is a rare, potentially fatal illness that affects less than 0.5% of individuals diagnosed with SLE [7,8]. Although the pentad of severe thrombocytopenia, fever, neurological problems, renal insufficiency, and microangiopathic hemolytic anemia was initially described in 1924, it was not until 1966 that it was published [9].

It is postulated that individuals who have acquired TTP as a result of SLE typically have lower levels of ADAMTS13 in their blood [7]. This is because they develop autoantibodies against the enzyme, which cleaves large precursors of VWF into smaller units that have less adhesive surface area for platelets. A high concentration of large multimeric forms of VWF accumulates in the blood due to ADAMTS13 deficiency, and these forms serve as strong adhesive surfaces for the development of platelet-rich thrombi that constrict the lumen of arterioles [8,9]. When red blood cells squeeze past these blood clots, they are under extreme stress, which damages their membranes and causes microangiopathic hemolysis [7].

This microthrombi can also harm and interrupt the blood flow, resulting in end-organ ischemia. Kumar et al. [10] have supported the theory of antibody-mediated ADAMTS13 degradation and have reported reduced blood ADAMTS13 levels in SLE patients. The validity of this theory is still unclear overall, but our example supports it further because our patient's blood ADAMTS13 levels were low when assessed during her hospital course.

The most common manifestations of the neuropsychiatric form of SLE (NP-SLE) continue to be headaches, psychoses, seizures, and cerebrovascular illnesses [11]. In SLE, the mechanisms that lead to the creation and buildup of immune complexes either directly harm the brain's neurons and arteries or indirectly cause damage to the central nervous system (CNS). The peripheral nervous system (PNS) and CNS are both involved in NP-SLE. Headache is the most common neuropsychiatric symptom. Lupus headaches cannot be effectively treated with conventional analgesic medication. Psychosis is also commonly diagnosed in people with NP-SLE. Cognitive impairment is a common symptom in NP-SLE patients, and symptoms might include coma, severe disorientation, and difficulties focusing. These are the people who often appear to be having seizures. More generalized seizures occur more frequently than focal seizures. Patients with NP-SLE may experience aberrant movement as well. Movement disorders include tremors, hemiballismus, cerebellar ataxia, chorea, and movements that mimic parkinsonian symptoms. Chorea is the most common movement issue and is more common in pediatric SLE than adult SLE. One of SLE's most well-known neuro-ocular symptoms is optic neuropathy [12].

Due to the complexity of identifying the source of our patient's hazy symptoms upon arrival and the necessity of conducting a thorough workup to establish a definitive diagnosis, the management of her condition became extremely challenging. The outcomes of the laboratory tests regularly directed our management strategy. The patient received intravenous steroids, ideally high-dose methylprednisolone, for three days after initial resuscitation for a stable hemodynamic state. Methylprednisolone is often preferred over other treatments for TTP due to its immunosuppressive properties, even if its usage as a first-line therapy lacks solid evidence [13]. The medication compromises immune system function, ultimately opposing the creation of antibodies and the subsequent destruction they mediate. Following the initiation of steroids, the subsequent step in our patient's medical care was to arrange for plasma exchange. This procedure, as demonstrated in the study conducted by Hassan et al. [14], is currently acknowledged as the most effective approach for treating TTP. At the same time, Blombery et al. [13] concluded that plasma exchange is the cornerstone of managing acquired TTP and recommended starting it even in situations where TTP is suspected. Following five rounds of plasma exchange, clinical improvement was observed in conjunction with steroid medication. After the progression of the disease was ultimately stopped, the patient was able to be discharged with only moderate neurological impairment.

## Conclusions

SLE is a multisystem connective disease that can manifest in rarer presentations, such as anemia and multiple strokes occurring simultaneously. The treating physician should keep these rare possibilities in mind while treating cases of SLE. This case describes a patient diagnosed with SLE with co-existing TTP, which was treated with hemodialysis and plasmapheresis. When SLE patients show signs of CNS involvement, it is important to evaluate the possibility of TTP as a potential cause. This is because TTP can mask a considerable secondary, life-threatening issue. General physicians should be aware of the fact that it was complicated to determine the source of this patient's hazy symptoms upon arrival, necessitating a thorough workup to establish a definitive diagnosis and management. The outcomes of the laboratory tests should direct the approach to managing these patients.
